# Selective targeting of *Scn8a* prevents seizure development in a mouse model of mesial temporal lobe epilepsy

**DOI:** 10.1038/s41598-017-17786-0

**Published:** 2018-01-09

**Authors:** Jennifer C. Wong, Christopher D. Makinson, Tyra Lamar, Qi Cheng, Jeffrey C. Wingard, Ernest F. Terwilliger, Andrew Escayg

**Affiliations:** 1 Department of Human Genetics, Emory University, Atlanta, Georgia 30322 USA; 2 Department of Medicine, Beth Israel Deaconess Medical Center, Harvard Medical School, Boston, MA 02215 USA

## Abstract

We previously found that genetic mutants with reduced expression or activity of *Scn8a* are resistant to induced seizures and that co-segregation of a mutant *Scn8a* allele can increase survival and seizure resistance of *Scn1a* mutant mice. In contrast, *Scn8a* expression is increased in the hippocampus following status epilepticus and amygdala kindling. These findings point to *Scn8a* as a promising therapeutic target for epilepsy and raise the possibility that aberrant overexpression of *Scn8a* in limbic structures may contribute to some epilepsies, including temporal lobe epilepsy. Using a small-hairpin-interfering RNA directed against the *Scn8a* gene, we selectively reduced *Scn8a* expression in the hippocampus of the intrahippocampal kainic acid (KA) mouse model of mesial temporal lobe epilepsy. We found that *Scn8a* knockdown prevented the development of spontaneous seizures in 9/10 mice, ameliorated KA-induced hyperactivity, and reduced reactive gliosis. These results support the potential of selectively targeting *Scn8a* for the treatment of refractory epilepsy.

## Introduction

Epilepsy is a common neurological disorder that affects approximately 50 million people worldwide^1^. As many as a third of these patients do not achieve adequate seizure control^2^. Unfortunately, the proportion of patients who fail to adequately respond to treatment has not substantially changed in decades. Thus, there is a clear need to develop new strategies that can effectively reduce seizure occurrence and the associated comorbidities for these patients while minimizing unwanted side effects.

*SCN8A*, which encodes the voltage-gated sodium channel (VGSC) Na_v_1.6, is broadly expressed throughout the central and peripheral nervous systems where it strongly modulates neuronal excitability by setting the threshold for action potential initiation and generating subthreshold depolarizing currents in the soma and dendrites^3^. In mice, heterozygous loss-of-function *Scn8a* mutations increase resistance to electrically (6 Hz) and pharmacologically (kainic acid, KA) induced seizures^4^ and amygdala kindling^5^, suggesting that *Scn8a*-conferred seizure protection is, in part, mediated by effects on the limbic system. The electrical induction of status epilepticus also leads to increased persistent and resurgent currents, which can be inhibited by the partially selective blockade of Na_v_1.6 using 4,9-anhydro-tetrodotoxin (4,9-ah-TTX)^6^. Strikingly, the seizure phenotypes and survival of *Scn1a* mutant mouse models of Dravet syndrome and GEFS+ can be dramatically improved by the co-expression of a heterozygous *Scn8a* loss-of-function allele^4,7^. Furthermore, *Cre*/*lox*P-mediated deletion of a floxed *Scn8a* allele in the hippocampus reduces pharmacologically-induced generalized tonic-clonic seizures *in vivo* ^8^. In contrast, prolonged increases in *Scn8a* expression are observed in the CA1 and CA3 regions of the hippocampus^9^ 60 days and two weeks following pilocarpine-induced status epilepticus (SE) and amygdala kindling^5^, respectively. An increase in *Scn8a* expression was also observed in the medial entorhinal cortex^6^ one week following electrically-induced SE. Na_v_1.6 expression is also increased in reactive astrocytes for up to 2 months following intrahippocampal KA injection in rats^10^. These findings raise the possibility that aberrant overexpression of *Scn8a* in limbic structures may be pathogenic in some types of epilepsy, including temporal lobe epilepsy (TLE), thereby pointing to *SCN8A* as a promising therapeutic target.

Temporal lobe epilepsy is the most common form of treatment-resistant adult epilepsy, and mesial temporal lobe epilepsy (MTLE) is the most common form of TLE^11^. MTLE is characterized by spontaneous seizures, behavioral abnormalities, and hippocampal pathology, including aberrant transcription, altered morphology, and neuroinflammation^12–15^. While some antiepileptic drugs used in the treatment of MTLE act, at least in part, on VGSCs, subtype-specific targeting has yet to be evaluated in preclinical studies. Furthermore, many of these antiepileptic drugs are not effective or can worsen clinical presentation in disorders such as Dravet syndrome, a severe early childhood encephalopathy that is commonly caused by mutations in the VGSC *SCN1A* (encoding Na_v_1.1)^16–18^.

It is increasingly recognized that the pathological consequences of altered VGSC activity are influenced by the different cell types and regions of the brain that are affected, which can also influence potential therapeutic options^19–22^. It is also evident that different VGSCs play distinct roles in pathology, yet therapeutically relevant strategies that take advantage of these findings have not been explored adequately. Here we examine the therapeutic potential of selectively reducing the expression of *Scn8a* in the hippocampus of a mouse model of MTLE using an adeno-associated viral vector (AAV) expressing a small-hairpin RNA construct against *Scn8a* (shRNA-Scn8a).

## Results

### Effective knockdown of *Scn8a* expression

**Figure 1:**
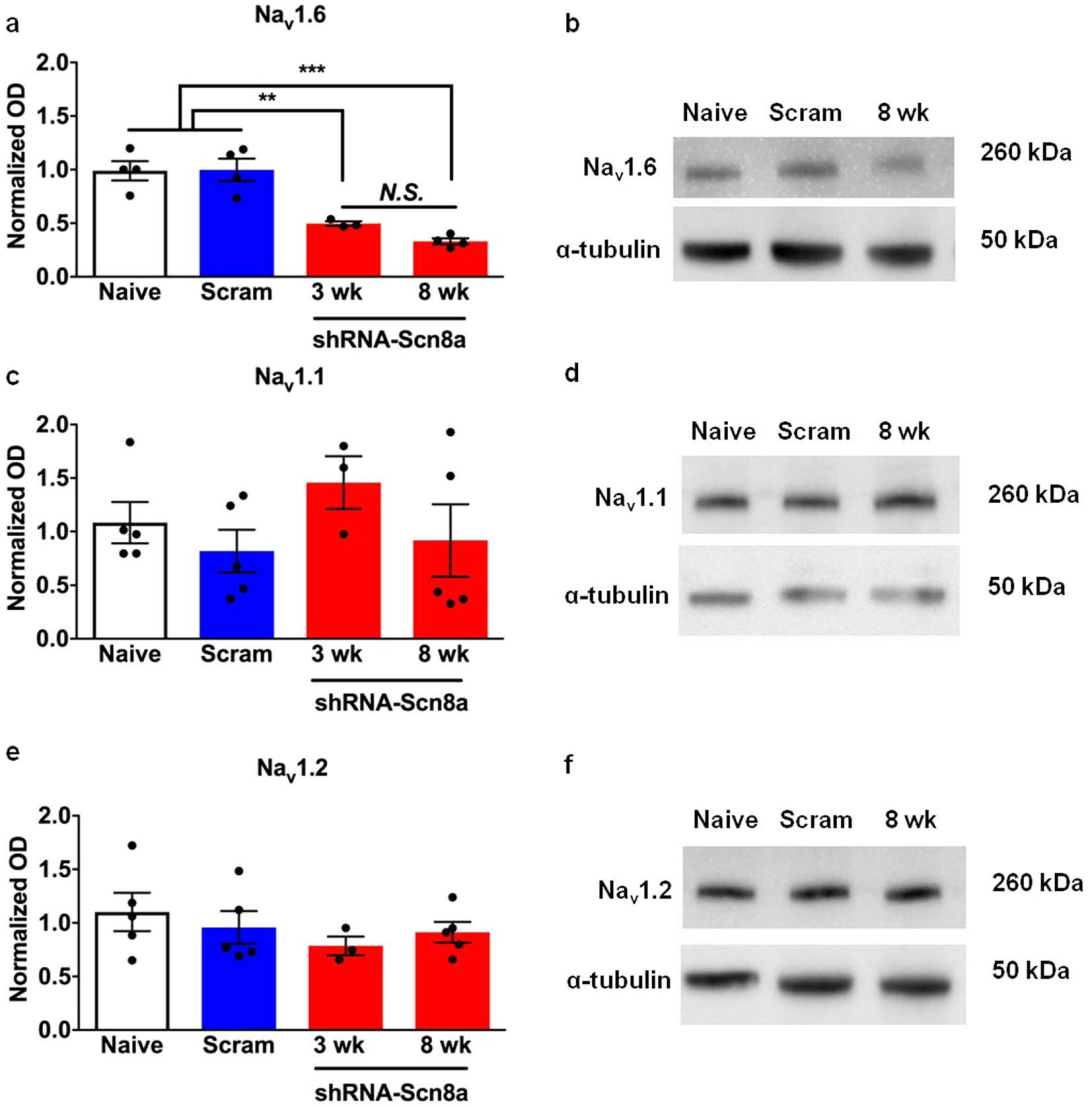
Effect of *Scn8a* knockdown on Na_v_1.6, Na_v_1.1, and Na_v_1.2 protein levels. **(a**,**b**) Protein expression of Na_v_1.6 was comparable between naïve mice and mice administered shRNA-scram. A significant reduction in protein expression levels was observed between naïve/shRNA-scram and shRNA-Scn8a-treated mice 3 weeks (*p* < 0.01) and 8 weeks (*p* < 0.001) following shRNA-Scn8a administration. Na_v_1.6 protein expression in shRNA-Scn8a-treated MTLE mice was not significantly different at 3 and 8 weeks following administration. One-way ANOVA followed by Bonferroni’s multiple comparisons test (*N* = 3–5/group). Protein expression levels of Na_v_1.1 (**c**,**d**) and Na_v_1.2 (**e**,**f**) were comparable across all groups. (**b**,**d**, and **f**) Representative cropped images of Western blot gel of each sodium channel and α-tubulin from hippocampi of naïve, shRNA-Scram-, and 8-week shRNA-Scn8a-injected mice. Full-length Western blot gels are presented in Supplementary Figure S1. All data are presented as mean ± SEM. **p < 0.01, ***p < 0.001.

To determine the efficiency and specificity of *Scn8a* knockdown by shRNA-Scn8a, we determined the expression of Na_v_1.1, Na_v_1.2, and Na_v_1.6 (encoded by the VGSC genes *SCN1A*, *SCN2A*, and *SCN8A*, respectively) 3 and 8 weeks following AAV administration. Naïve and shRNA-scram injected mice had comparable protein levels of each VGSC. We observed reductions of approximately 50% and 70% in Na_v_1.6 after 3 (*p* < 0.01) and 8 weeks (*p* < 0.001) following shRNA-Scn8a administration, respectively (Fig. 1a,b, one-way ANOVA followed by Bonferroni’s multiple comparisons test). Comparable levels of Na_v_1.1 and Na_v_1.2 were observed across all groups (Fig. 1c–f).

### *Scn8a* knockdown confers robust protection against spontaneous seizures in the MTLE model

**Figure 2:**
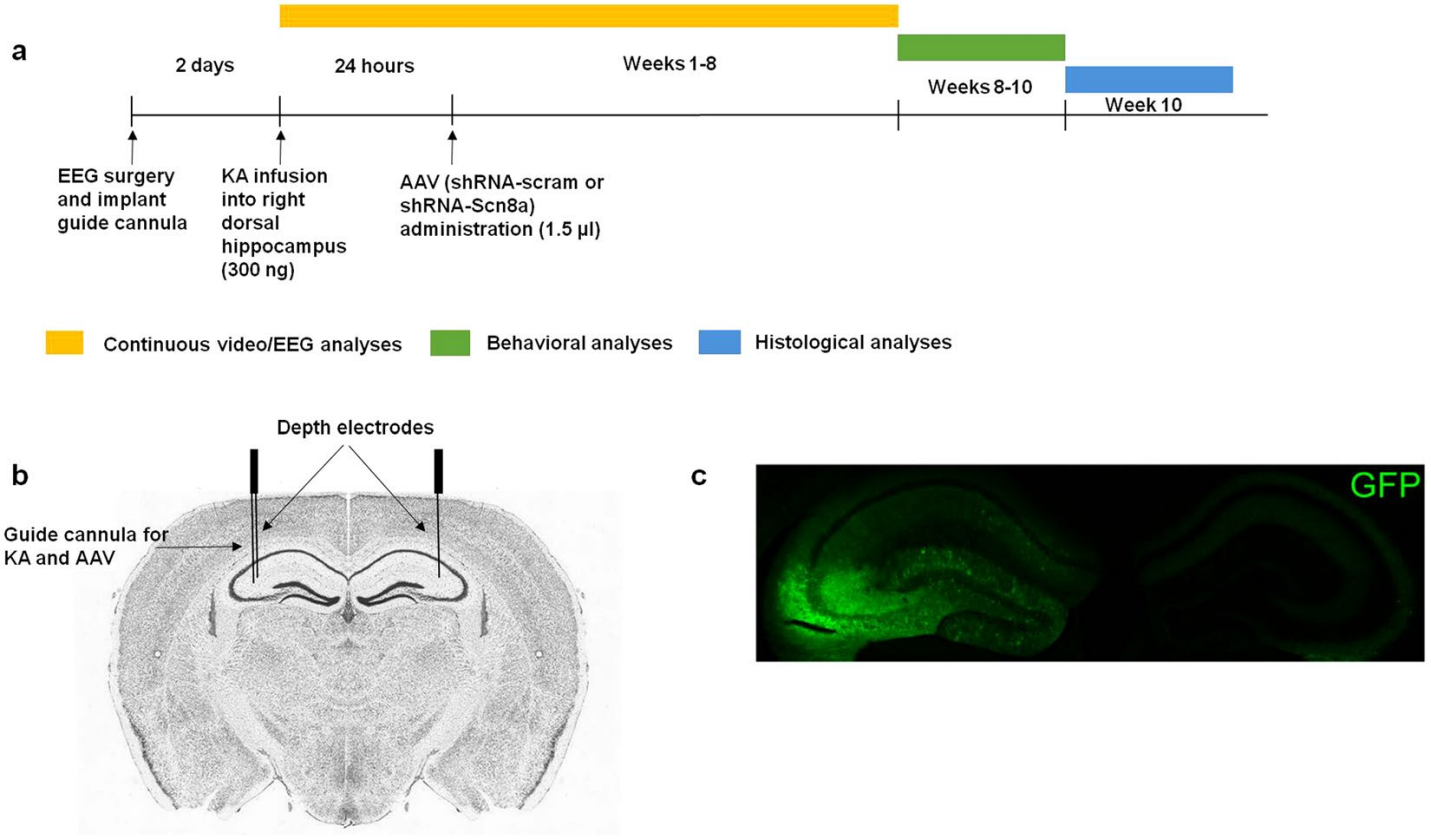
Schematic of experimental design. (**a**) Schedule of treatment, EEG, behavioral, and histological analyses. Mice were administered KA, followed by AAV constructs 24 hours later (post-status epilepticus). Continuous video/EEG analyses were performed for 8 weeks, followed by 2 weeks of behavioral analyses, and histological analyses at week 10 post-KA. (**b**) Depth EEG recording electrodes were placed in the dorsal hippocampus (coordinates relative to bregma and from the surface of the brain: (AP) −1.8 mm, (ML) ± 1.6 mm, (DV) −1.7 mm). A guide cannula (for KA and AAV administration) was attached laterally to the depth electrode in the right dorsal hippocampus. Brain image adapted from Allen Brain Atlas. (**c**) Representative image of hippocampus and GFP expression.

We first examined the effect of *Scn8a* knockdown in the mouse model of MTLE in which the hippocampus is the primary site of initial seizure generation (Fig. 2a). Male C57BL/6 mice were implanted with depth and cortical electrodes (Fig. 2b). Following a 2-day recovery period, we injected the mice with KA directly into the right dorsal hippocampus via a cannula. Twenty-four hours after KA administration, shRNA-Scn8a or shRNA-scram were similarly introduced into the hippocampus using the cannula. GFP expression was imaged at the end of the experiment to confirm that the right dorsal hippocampus was targeted (Fig. 2c).

**Figure 3:**
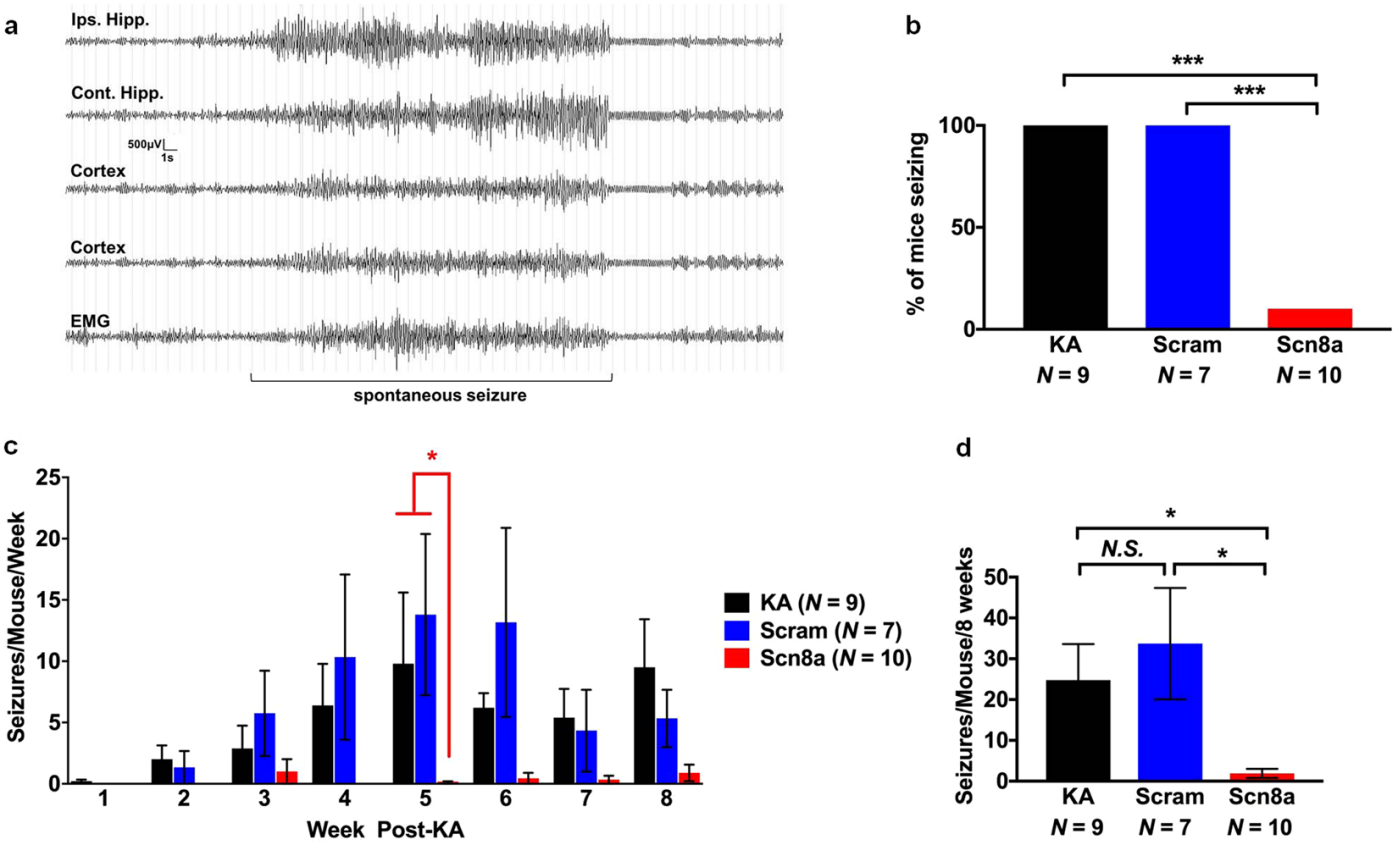
*Scn8a* knockdown provides robust seizure protection in a mouse model of MTLE. (**a**) Representative EEG traces from two depth electrodes in the dorsal hippocampus, two cortical electrodes, and EMG before, during, and after a spontaneous seizure. (**b**) Percent of mice seizing during the 8 weeks of EEG analyses. Only 1/10 (10%) of shRNA-Scn8a-treated MTLE mouse developed spontaneous seizures whereas all KA-only and shRNA-scram-treated MTLE mice exhibited seizures. The data for the single shRNA-Scn8a-treated mouse that exhibited spontaneous seizures is divided by 10 to obtain the average values shown in panels c and d. (**c**) Number of seizures weekly was dramatically lower in shRNA-Scn8a-treated MTLE mice compared to KA only and shRNA-Scram-treated MTLE mice. (**d**) Average number of seizures during the 8 weeks of the EEG recording period was comparable between KA-only and shRNA-scram-treated mice. The average number of seizures in shRNA-Scn8a-treated mice was significantly lower than shRNA-scram (*p* < 0.05) and KA-only (*p* < 0.05) mice. *N* = 7–10/group. Non-parametric Kruskal-Wallis test followed by Dunn’s multiple comparisons test. All data are presented as mean ± SEM. *p < 0.05, ***p < 0.001, *N.S*, not significant.

The latency to the first spontaneous seizure between the one shRNA-Scn8a-treated MTLE mouse that seized (14 days to first seizure) was not significantly different from the KA-only (11.3 ± 1.4 days) and shRNA-scram-treated MTLE mice (13.7 ± 0.3 days). The average number of spontaneous seizures observed each week was comparable between KA-only and shRNA-scram-treated mice; however, we saw a dramatic reduction in the number of spontaneous seizures in shRNA-Scn8a-treated MTLE mice compared to controls at all time points (Fig. 3c). Of the shRNA-Scn8a treated MTLE mice, 90% (9/10) were seizure-free during the 8-week EEG recording period, whereas all controls (KA-only and shRNA-scram-treated MTLE mice) exhibited spontaneous seizures (Fig. 3b). The average number of spontaneous seizures during the 8-week period was significantly lower in the shRNA-Scn8a-treated MTLE mice compared to shRNA-scram (*p* < 0.05) and KA-only-treated mice (*p* < 0.05, non-parametric Kruskal-Wallis test followed by Dunn’s multiple comparisons test, Fig. 3d). Seizure length (30–40 seconds) was found to be comparable among the control groups and the few seizures observed in the one shRNA-Scn8a-treated MTLE mouse that exhibited spontaneous seizures. Regardless of treatment, all electrographic seizures were accompanied by behavioral generalized tonic-clonic seizures characterized by rearing, paw waving, head bobbing, and loss of posture as evidenced by simultaneous video analyses (Supplemental video S1).

### Reduced *Scn8a* expression ameliorates hyperactivity in the MTLE model

Patients with MTLE often exhibit increased anxiety, impaired learning and memory, and hippocampal neuronal loss^12–14,23–25^. Similar abnormalities in the intrahippocampal KA mouse model have also been reported in some studies^26–29^. To determine whether reduced *Scn8a* expression could also ameliorate the anticipated behavioral abnormalities, we conducted behavioral assessments upon completion of the 8 weeks of continuous video/EEG recordings. To control for any effect of the surgical procedure on behavior, we also generated mice that underwent the same surgical procedure but did not receive KA or AAV constructs (surgery-only group, *N* = 10).

**Figure 4:**
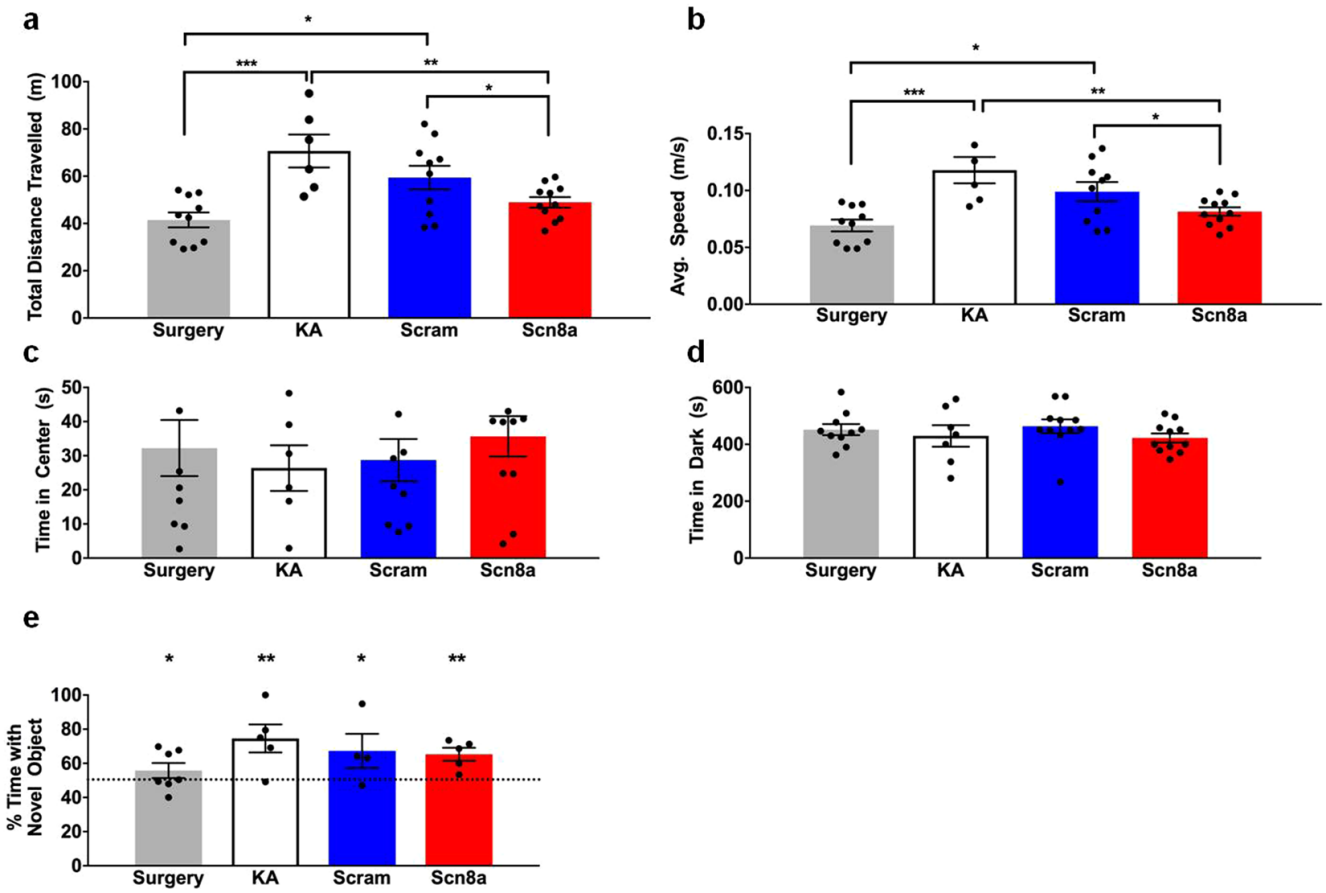
*Scn8a* knockdown reduces KA-induced hyperactivity. (**a**,**b**) Distance travelled and average speed were significantly higher in KA-only mice and mice treated with shRNA-scram when compared to shRNA-Scn8a-treated and surgery-only mice. (*N* = 6–11/group). (**c**) Time spent in center (open field paradigm) and (**d**) time spent in the dark (light/dark box paradigm) were comparable between all groups of mice. (*N* = 6–11/group). One-way ANOVA followed by the Holm-Sidák’s multiple comparisons test. (**e**) Percent of time spent exploring the novel object was not significantly different between all groups but was significantly greater than 50% chance. (*N* = 4–7/group). One-tailed student’s *t* test. All data are presented as mean ± SEM. *p < 0.05, **p < 0.01, ***p < 0.001.

In the open field paradigm, we found that KA-only and shRNA-scram-treated mice travelled at significantly higher speeds and covered greater distances during the 10-minute period compared to the shRNA-Scn8a-treated MTLE mice and surgery-only controls (Fig. 4a,b). Speed and distance travelled were comparable between the shRNA-Scn8a-treated MTLE mice and the surgery-only group, but both groups were statistically different from the KA-only and shRNA-scram-treated mice, suggesting that reduced *Scn8a* expression can ameliorate KA-induced hyperactivity. Anxiety levels, as assessed by the open field and light/dark box paradigms, were similar for all groups of mice (Fig. 4c,d). Learning and memory was assessed using the novel object recognition (NOR) paradigm (Fig. 4e). Although some variability was observed between the different groups of mice, within each group, more than 50% of the time was spent exploring the novel object, consistent with preference of mice for a novel versus familiar object, and indicative of normal learning and memory (*p* < 0.05 surgery only and shRNA-scram-treated MTLE mice, *p* < 0.01 KA only and shRNA-Scn8a-treated MTLE mice, one-tailed student’s *t* test, Fig. 4e).

### Knockdown of *Scn8a* in the hippocampus reduces GFAP expression but not neuronal loss in the MTLE model

**Figure 5:**
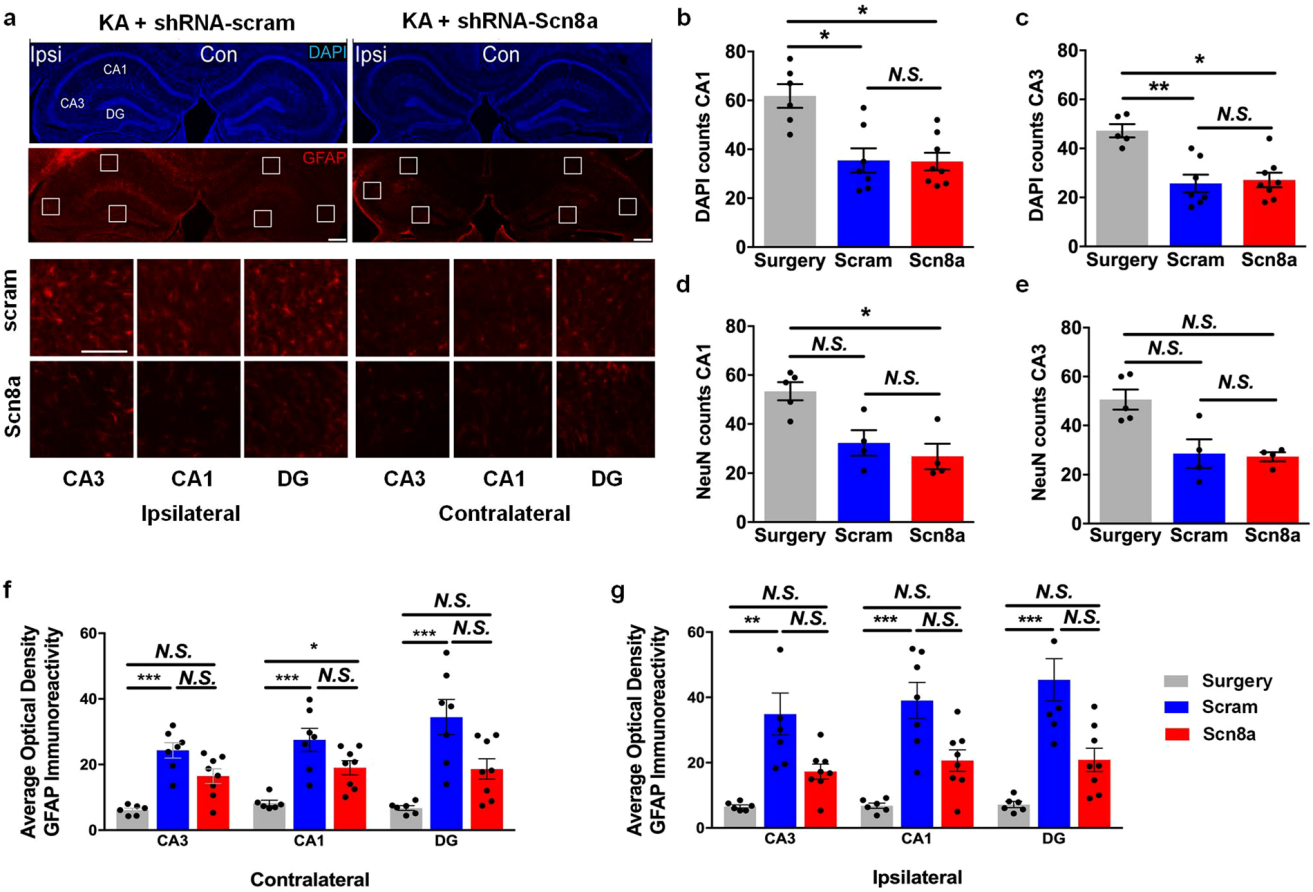
*Scn8a* knockdown reduces GFAP expression in a mouse model of MTLE. (**a**) Representative images of hippocampi following kainic acid (KA) and either control shRNA-scram (left column) or shRNA-Scn8a (right column) injections. DAPI staining (top) shows the hippocampal ultrastructure. GFAP immunoreactivity (bottom) was used to compare the extent of reactive gliosis in AAV-treated MTLE mice. Scale bar, 300 µm. Inset images show CA3, CA1, and DG regions of the ipsilateral (left panel) and contralateral (right panel) hippocampi following treatment with either shRNA-scram (top row) or shRNA-Scn8a (bottom row). Scale bars, 100 µm. Approximately 50% reduction in number of cells (**b**,**c**) and neurons (**d,e**) in the ipsilateral hippocampus were observed in MTLE animals treated with either shRNA-scram (blue) or shRNA-Scn8a (red) compared to surgery-only controls (gray). (**f**,**g**) Quantification of optical density (O.D.) of normalized GFAP immunoreactivity in the contralateral and ipsilateral hippocampi in surgery-only (gray) or MTLE animals treated with either shRNA-scram (blue) or shRNA-Scn8a (red). GFAP expression was comparable between the surgery-only controls (gray) and shRNA-Scn8a-treated MTLE mice (red) in the ipsilateral hippocampus. In contrast, GFAP expression was significantly increased in the shRNA-scram-treated MTLE mice compared to surgery-only controls in both the ipsilateral and contralateral hippocampus. Non-parametric Kruskal-Wallis test followed by Dunn’s multiple comparisons test (NeuN: *N* = 4–5/group, DAPI and GFAP: *N* = 6–8/group). All data are presented as mean ± SEM. *p < 0.05, **p < 0.01, ***p < 0.001, *N.S*, not significant.

To determine whether reduced *Scn8a* expression could protect against hippocampal alterations in the MTLE model, we compared neuronal and cell loss and reactive gliosis between surgery-only mice and MTLE mice treated with shRNA-scram or shRNA-Scn8a. In the CA3 and CA1 regions of the contralateral hippocampus, cell and neuron counts as measured by DAPI (Fig. 5a) and NeuN staining (not shown), respectively, were comparable across the three groups of mice. However, in the ipsilateral CA3 and CA1 regions, DAPI (Fig. 5b,c) and NeuN (Fig. 5d,e) staining revealed approximately 50% cell and neuron loss, respectively, in MTLE mice regardless of treatment when compared to surgery-only controls.

In the contralateral CA3, and DG regions, GFAP immunoreactivity was not significantly different between shRNA-Scn8a-treated MTLE and surgery-only mice; however, GFAP expression was significantly higher in the contralateral CA3, CA1 and DG regions of shRNA-scram-treated MTLE mice compared to surgery-only controls (Fig. 5a,f). In the ipsilateral CA3, CA1, and DG regions, we observed comparable GFAP expression between the surgery-only controls and shRNA-Scn8a-treated MTLE mice, while GFAP expression in the shRNA-scram-treated MTLE mice was significantly higher compared to surgery-only controls (non-parametric Kruskal-Wallis test followed by Dunn’s multiple comparisons test, Fig. 5g). These results demonstrate that while *Scn8a* knockdown did not protect against cell and neuron loss in the ipsilateral hippocampus of the MTLE mice, it did result in significantly less reactive gliosis throughout the hippocampus.

## Discussion

The first human epilepsy mutation in *SCN8A* was identified in 2012^30^, and since then, there have been a number of additional *SCN8A* mutations reported^31–36^. On the basis of electrophysiological analyses using heterologous systems, *SCN8A* mutations associated with epileptic encephalopathy typically result in alterations such as increased persistent current or slower channel inactivation^30,32,35^, which are predicted to increase channel activity. For example, spontaneous firing and an increased number of action potentials were observed in dissociated hippocampal neurons expressing the *SCN8A* epilepsy mutation Asn1768Asp^30^. In contrast, we previously demonstrated that mutant mice with reduced *Scn8a* expression (*Scn8a* ^Med/+^) have increased seizure resistance and the seizure phenotype of *Scn1a* mutant mice could be ameliorated by the co-expression of a mutant *Scn8a* allele^4,7,37^. Heterozygous *Scn8a* null mice have an increased latency to initiate hippocampal epileptiform burst activity and reduced burst discharge activity when compared to WT littermates^8^.

A previous study by Zhu and colleagues demonstrated that *Scn8a* mRNA expression was significantly increased in the ipsilateral hippocampus for up to 2 months following intrahippocampal KA-induced status epilepticus in rats, and the magnitude of the increase was correlated with the severity of SE^10^. In addition, increased *Scn8a* mRNA and protein levels were also observed in the hippocampus^9^ following systemic pilocarpine-induced SE. These observations provide support for the therapeutic potential of selective *Scn8a* knockdown in the MTLE model. In contrast, Qiao *et al*.^38^ observed reduced sodium channel expression (Na_v_1.1, Na_v_1.2, and Na_v_1.6) in neurons surviving systemic KA-induced SE; however, Na_v_1.6 levels were elevated in astrocytes at 3 weeks following SE.

Since pharmacological compounds that can selectively target *Scn8a* are not clinically available yet, we used an AAV-shRNA to investigate the effect of selective hippocampal knockdown of *Scn8a* expression in a mouse model of MTLE. Using a single injection of shRNA-Scn8a into the hippocampus 24 hours after KA administration, we found that we could effectively abolish the development of spontaneous seizures, mitigate KA-induced hyperactivity, and reduce reactive gliosis. To our knowledge, this is the first study to show that reducing *Scn8a* expression can prevent the onset of spontaneous seizures in a mouse model of MTLE.

The latent period following KA-induced SE is characterized by dramatic changes to neural networks in the limbic system that ultimately lead to the generation of spontaneous seizures^39^. It was initially presumed that dispersion of granule cells and mossy fiber sprouting initiated epileptogenesis during the latent period^40^. However, blockade of mossy fiber sprouting by cycloheximide administration did not inhibit the development of spontaneous seizures in the intrahippocampal KA model of MTLE^41^. Since considerable cell and neuronal loss and the dispersion of granule cells in the ipsilateral dorsal hippocampus can be detected during the latent period^42^, preventing these histological changes may prevent or alter epileptogenesis. In addition to histological changes within the hippocampus, there are also distinct alterations in electrographic activity. Within the 2 weeks following SE, bursts of high frequency, low voltage discharges are observed in the ipsilateral hippocampus. Thereafter, the chronic epileptic period is characterized by high frequency bursts and high voltage sharp waves in addition to spontaneous seizures^39^. As such, the latent period might provide a critical window during which interventions may be most effective at preventing or mitigating the development of spontaneous recurrent seizures.

Liu and colleagues^43^ previously found, using the intra-amygdala MTLE model, that inhibition of the brain-derived neurotrophic factor receptor TrkB during the latent period provided protection against the development of spontaneous recurrent seizures, anxiety-like behavior, and hippocampal neuron loss. Consistent with the findings of Liu *et al*.^43^, we found that, in the absence of intervention, spontaneous seizures began approximately 2 weeks following KA administration. However, administration of shRNA-Scn8a 24 hours following KA administration effectively abolished the onset of spontaneous seizures in 90% (9/10) of mice (Fig. 3b). Although later intervention time points remain to be tested, these results demonstrate that early *Scn8a* knockdown may interrupt epileptogenesis, providing robust seizure protection.

Patients with MTLE often exhibit a range of behavioral deficits, including increased anxiety and impaired learning and memory^24,25^. Although the intrahippocampal KA model of MTLE recapitulates several of the behavioral characteristics of MTLE patients, there are inconsistencies between studies. For example, Groticke *et al*.^26^ observed normal anxiety levels in MTLE mice when assessed with the elevated plus maze and light/dark box paradigms whereas O’Loughlin *et al*.^27^ found increased anxiety in MTLE mice using the elevated plus maze. In our hands, we found similar anxiety levels across all groups as reflected in the time spent in the center of the open field and dark side of the light/dark box (Fig. 4c,d). Increased locomotor activity in the MTLE mouse model during the habituation phase of novel object recognition has previously been reported^26^, suggesting a hyperactive phenotype. Similarly, we observed hyperactivity in the MTLE model as evidenced by increased locomotor activity in the KA-treated mice (Fig. 4a,b). Moreover, we found that hippocampal *Scn8a* knockdown was sufficient to restore locomotor activity to the level of surgery-only control mice. Consistent with the findings of Groticke *et al*.^26^, we did not observe learning and memory deficits in the MTLE mice. Importantly, reducing *Scn8a* expression in the hippocampus did not lead to increased anxiety levels or impaired learning and memory.

Changes in hippocampal morphology, including neuronal loss and reactive gliosis, is often observed in MTLE patients^44^ and is recapitulated in the intrahippocampal KA model of MTLE^26,39^. Groticke *et al*.^26^ observed significant neuronal degeneration in the ipsilateral CA1 and CA3 regions in MTLE mice, with the most pronounced degeneration in the CA3 region, whereas no significant neuronal loss was observed in the contralateral hippocampus. We similarly observed significant cell and neuron loss in the ipsilateral CA1 and CA3 regions of both shRNA-Scn8a- and shRNA-scram-treated MTLE mice, indicating that *Scn8a* knockdown is not protective against this component of the phenotype. Our inability to protect against cell loss is not surprising given that significant cell and neuron loss has been observed in the CA1 and CA3 regions as early as 2 hours post-KA administration^29^, and the level of AAV-mediated gene knockdown increases during the initial weeks following administration^45^. Since reduced *Scn8a* expression did provide robust protection against spontaneous seizures and hyperactivity, our results suggest that neuronal protection is not required for seizure protection or for the amelioration of at least some behavioral phenotypes.

Contrary to the lack of *Scn8a-*mediated protection against cell and neuronal loss, we did, however, observe reduced GFAP immunoreactivity, a marker of gliosis^46^, in the shRNA-Scn8a-treated MTLE mice compared to shRNA-scram-treated mice. In a systemic mouse model of pilocarpine-induced SE, GFAP immunoreactivity was not detected in the hippocampus until at least 12 hours following SE and progressively increased until 1 week following SE^47^. In the intrahippocampal KA mouse model, GFAP immunoreactivity was detected 4 days following SE and gradually increased until 30 days following SE^48^. These findings demonstrate that the temporality of reactive gliosis reflects the impact of CNS insult, whereby the larger the insult (systemic pilocarpine) results in reactive gliosis sooner than following a focal insult (intrahippocampal KA). Furthermore, these findings also demonstrate a slower progression of reactive gliosis compared to neuronal degeneration which occurs shortly following SE. Reactive gliosis is a dynamic process following CNS insult that includes many types of glia, including oligodendrocytes, microglia, and astrocytes^46,49^. GFAP mRNA and protein levels are known to be elevated following both electrically- and chemically-induced seizures^50^, and repeated seizures also result in reactive gliosis^51^. In addition, it was recently reported that an *Scn8a* mouse model of encephalopathy (characterized by elevated mutant Na_v_1.6 channel activity) exhibits increased hippocampal reactive astrocytosis following the development of spontaneous seizures^52^. In the present study, it is likely that selective reduction in *Scn8a* expression would have directly decreased neuronal excitability, thereby conferring seizure protection in shRNA-Scn8a treated MTLE mice^8,53^. In turn, the lower level of seizure activity in shRNA-Scn8a treated MTLE mice was reflected in reduced reactive gliosis compared to shRNA-scram-treated MTLE mice that experienced ongoing seizure activity.

Overall, we demonstrated that a selective reduction in *Scn8a* can dramatically protect against the development of spontaneous seizures, hyperactivity, and reactive gliosis in a mouse model of MTLE. These findings highlight the protection that can be conferred by reducing *Scn8a* expression in models of treatment-resistant epilepsy.

## Materials and Methods

### Animals

Male C57BL/6 mice (Charles River, 027, 8–10 weeks old) were used to generate the mouse model of MTLE. After surgery, all mice were single housed in a clear Plexiglas cage (8.5 × 8.5 × 12.25 inches) on a 12-h light/dark cycle, with food and water available *ad libitum*. All experiments were performed in accordance with the National Institutes of Health guidelines for the care and use of laboratory animals under protocols approved by the Institutional Animal Care and Use Committee of Emory University.

### *Scn8a* knockdown

#### Development of the shRNA construct

We developed an AAV construct containing an shRNA against the mouse *Scn8a* gene (shRNA-Scn8a, 5′- AAGCTGTCAGTCGTGATGATC TTCAAGAGA GATCATCACGTCTGACAGCTT −3′, 21-mer mismatch sense + 9-mer linker + 21-mer antisense)^53^. We also generated a control construct (shRNA-scram) with a scrambled sequence (AGTACTGCTTACGATACGG) that does not correspond to any known mouse mRNA sequence. AAV10 was selected because it preferentially targets and transduces neurons when injected directly into the brain without transducing glia^54^ and is known to have a considerably faster rate of action than AAV2, with reporter expression observable at approximately 10 days following administration^45^. AAV cloning and packaging was performed as previously described^45^. Briefly, a synthetic oligo, corresponding to either the mouse *Scn8a* cDNA or scrambled sequence, was annealed and ligated into unique *Bbs*l and *Nhe*l sites in an EGFP-U6-AAV plasmid downstream of the U6 promoter. The EGFP was driven by a cytomegalovirus (CMV) promoter followed by a SV40 intron/polyA sequence. Packaging was performed using a standard triple transfection protocol to generate helper virus-free pseudotyped AAV10 virus. The average titer of each preparation was approximately 1 × 10^12^ vector genomes/ml.

### Time course of *Scn8a* knockdown

shRNA-Scn8a and shRNA-scram were each injected into the hippocampus (1.5 µL/site) of male C57BL/6 mice (*N* = 3–5/group) at optimized coordinates, relative to bregma and from the surface of the brain: (AP) −2.0 mm, (ML) ± 1.5 mm, (DV) −1.5 mm. Posterior hippocampal injection sites were: (AP) −3.0 mm, (ML): ± 3.0 mm, (DV) −3.0 mm. Four injections (two each into the dorsal and posterior hippocampus) were performed to maximize targeting of the hippocampus and to achieve sufficient knockdown of *Scn8a* for quantification by Western Blot analyses. Naïve mice (*N* = 5) that did not receive AAV constructs were used as controls. Mice were sacrificed at 3 and 8 weeks after AAV administration, their hippocampi dissected, and protein isolated.

### Western blot analysis

Isolated hippocampal protein samples from each mouse (15–25 µg) were loaded into each well of a gel (4–15% Mini-PROTEAN Precast protein gels, Biorad). Blots were probed with anti-Na_v_1.6 (1:200; Millipore, Billerica, MA), anti- Na_v_1.1 (1:200; Millipore), anti- Na_v_1.2 (1:200; Millipore), and mouse anti-α-tubulin antibodies followed by incubation with a secondary antibody (HRP-conjugated donkey anti-rabbit for sodium channels and HRP-conjugated goat anti-mouse for α-tubulin). Band intensities were analyzed with ImageJ software (NIH) and normalized to α-tubulin.

### Generation of a mouse model of MTLE

Male C57BL/6 mice (10 weeks old) were anesthetized with isoflurane and surgically implanted with a 23-gauge injection guide cannula attached to an EEG recording electrode for the delivery of reagents to the hippocampus and collection of EEG signals, respectively. Two bipolar depth electrodes (Plastics One) were implanted bilaterally into the dorsal hippocampus, and monopolar electrodes were placed over the left and right frontoparietal cortex at these coordinates relative to bregma: (AP): 2.0 mm, (ML) ± 1.5 mm. The guide cannula was unilaterally positioned on top of the dura and affixed to the depth electrode in the right dorsal hippocampus at these coordinates relative to bregma and from the surface of the brain: (AP) −1.8 mm, (ML) ± 1.6 mm, (DV) −1.7 mm. Two fine wires were implanted into the neck muscle to obtain electromyography (EMG) recordings. After allowing the mice to recover from surgery for 2 days, KA (300 ng) dissolved in 0.5 µL 1x phosphate buffered solution was injected unilaterally into the right dorsal hippocampus over a 4-minute period using a 30-gauge needle connected to a 5 μl Hamilton syringe. Sham surgery mice that did not receive KA or AAV constructs were also generated to control for the surgical procedure. Consistent with published data^29,39,55^, the KA-injected mice entered a period of SE which lasted for at least 3 hours. Mice that did not enter SE for at least 3 hours were excluded from analyses. This was followed by the development of high-voltage sharp waves in the ipsilateral (KA-injected) hippocampus as well as spontaneous seizures approximately 2 weeks after KA administration.

### Hippocampal *Scn8a* knockdown

#### Mouse model of MTLE

To determine whether reducing *Scn8a* expression in the hippocampus could prevent or ameliorate spontaneous seizures and behavioral abnormalities in the mouse model of MTLE, AAV constructs (shRNA-Scn8a or shRNA-scram) were administered (1.5 μl over a 4-minute period) into the right dorsal hippocampus through the implanted guide cannula 24 hours following KA administration (Fig. 2).

### EEG analyses

A detailed profile of electrographic activity was obtained from the MTLE mice by performing continuous video/EEG analyses (24 hours/day) following KA administration and continuing for 8 weeks. Seizure frequency and duration were recorded for each mouse during the 8-week period. Mice were tethered via a commutator (Dragonfly) during continuous video/EEG recordings, and electrographic signals were collected, processed, and analyzed with the Harmonie rodent software (Stellate). EEG/EMG signals were analyzed using a high-pass filter of 5 Hz, a low pass filter of 70 Hz, and a notch filter of 60 Hz. The left frontal cortical electrode was used as a reference for EEG/EMG signals. Seizures were characterized by the onset of high frequency and amplitude waves that were at least twice the background for all EEG electrodes, lasting for at least 10 seconds in duration (Fig. 3a). Seizures were manually identified and scored for frequency and duration by an experimenter who was blind to treatment group. Mouse behavior during electrographic seizures was confirmed by simultaneous video recordings (Supplemental video S1).

### Behavioral analyses

Behavioral analyses were conducted on all MTLE mice and controls after completion of 8 weeks of continuous video/EEG analyses. To assess hyperactivity, anxiety, and learning and memory, we used three well-established paradigms: open field^26,43^, light/dark box^26,43^, and novel object recognition using a two-object design^56^. Behavioral assessments were conducted under the same lighting conditions (475 lux) for each task. All mice were subjected to each behavioral task with a 1-week interval between each task.

#### Light/dark box

The light/dark box was constructed of plexiglas with separate light (20 × 14 × 14.5 cm; 475 lux) and dark sides (10 × 14 × 14.5 cm; 0 lux). Each mouse was placed in the center of the light side and allowed to freely explore the box for 10 minutes. The amount of time spent in the dark side and the number of transitions between the light and dark sides were recorded.

#### Open field and novel object recognition

Open field analyses were conducted on Day 1 of the novel object recognition task as previously described^37,57^. Briefly, on Day 1, each mouse was placed into the apparatus (60 × 60 × 60 cm; 475 lux) and allowed to freely explore for 10 minutes. Distance travelled, average speed, and the time spent in the center were recorded for each mouse. On Days 2 and 3 of novel object recognition, each mouse was allowed to freely explore the same two objects (either two spheres or two squares) for 10 minutes. On Day 4 of novel object recognition, one of the objects was replaced with a novel object (either a sphere or square), and each mouse was allowed to freely explore for 10 minutes. Objects were randomized for each mouse. A mouse that received two spheres on Days 2 and 3, received a square as the novel object on Day 4. Another mouse would receive two squares on Days 2 and 3, and a sphere as the novel object on Day 4. The percentage of time exploring the novel object (out of total object exploration time on Day 4) was recorded for each mouse.

### Histological analyses

Histological analyses were conducted in a subset of MTLE mice (NeuN: 4–5/group; DAPI and GFAP: *N* = 6–8/group) following the completion of behavioral analyses (10 weeks post-KA administration). Mice were sacrificed under deep isoflurane anesthesia and transcardially perfused with 4% paraformaldehyde. Brains were collected and post-fixed in 4% paraformaldehyde for 2 hours at 4 °C, and then placed into a 30% sucrose solution at 4 °C. Coronal slices (45 µm thick) were cryosectioned and preserved at −80 °C in cryoprotectant. Immunodetection procedures were followed as previously described^58^. Free-floating sections were incubated with the following primary antibodies: Polyclonal rabbit anti-GFAP (1:1000, Abcam), monoclonal mouse anti-Iba1 (1:500, Abcam), and polyclonal rabbit anti-NeuN (1:500, EMD Millipore). The following fluorescent secondary antibodies were used: Alexa fluor 594 goat anti-rabbit (1:1000, Invitrogen), and Alexa fluor 594 goat anti-mouse (1:1000, Invitrogen). Primary antibodies were incubated for 2 days at 4 °C while secondary antibodies were incubated for 2 hours at 23 °C. Slices were mounted using media that contained DAPI (Vector Laboratories) and imaged using a spinning disk confocal microscope (Zeiss motorized Axiovert 200 M) and Metamorph imaging software. All images for quantification were collected using the same microscope settings in the same sitting and processed simultaneously. Slices from each animal containing ipsilateral and contralateral dorsal hippocampi between the coordinates (relative to bregma) −1.40 mm to −2.20 mm, were analyzed. All quantification within each hippocampal region was conducted within a select frame (600 µm × 600 µm) averaged from at least 2 slices for each animal. Within the pyramidal cell layer, regions of interest containing CA1, CA3 and DG were chosen and analyzed by an investigator blinded to the treatment groups. All images were quantified using MetaMorph Microscopy Image Analysis Software (Molecular Devices) and ImageJ software (NIH).

### Statistical analysis

One-way ANOVA followed by Bonferroni’s multiple comparisons test was used to compare normalized protein levels from Western Blots of naïve and AAV-treated mice. A non-parametric Kruskal-Wallis test followed by Dunn’s multiple comparisons test was used to compare the total number of seizures during the 8-week EEG recording period between KA-, shRNA-scram-, and shRNA-Scn8a-treated mice. The Kruskal-Wallis test followed by Dunn’s multiple comparisons was also used to compare NeuN, DAPI, and GFAP counts between surgery-only, shRNA-scram-, and shRNA-Scn8a treated MTLE mice. One-way ANOVA followed by the Holm-Sidák’s multiple comparisons test was used to compare total distance travelled and average speed between surgery-only, KA-, and AAV-treated MTLE mice. All error bars represent ± the standard error of the mean. All data analyses were conducted by an experimenter blinded to treatment group, and all mice were randomly assigned to a treatment group.

## Electronic supplementary material

Supplementary information - Representative full-length Western blot gel images of Nav1.6, Nav1.1, and Nav1.2.

Example of EEG traces and simultaneous video recording before, during, and after a spontaneous seizure.
