# Supplementary material for: Selective targeting of Scn8a prevents seizure development in a mouse model of mesial temporal lobe epilepsy
Source: Sci Rep. 2018 Jan 9;8:126. doi: 10.1038/s41598-017-17786-0 (PMC5760706; doi:10.1038/s41598-017-17786-0)
Supplement: Supplementary file 1 — Supplementary information - Representative full-length Western blot gel images of Nav1.6, Nav1.1, and Nav1.2. [file 41598_2017_17786_MOESM1_ESM.doc]

Selective targeting of *Scn8a* prevents seizure development in a mouse model of mesial temporal lobe epilepsy

Jennifer C. Wong^a^, Christopher D. Makinson^a^, Tyra Lamar^a^, Qi Cheng^a^, Jeffrey C. Wingard^b^, Ernest F. Terwilliger^b#^, and Andrew Escayg^a^*


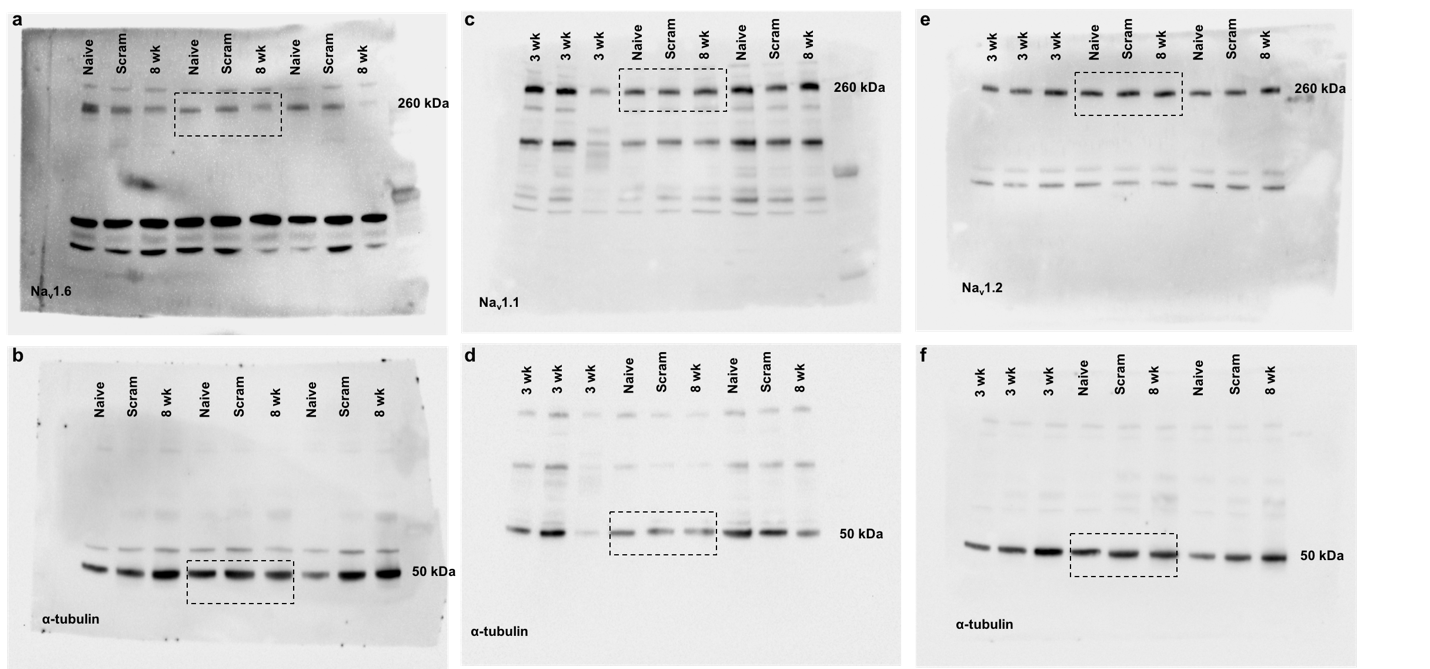


**Supplemental Figure S1. Representative full-length Western blot gel images of Na_v_1.6 (a-b), Na_v_1.1 (c-d), and Na_v_1.2 (e-f).** The dashed lines represent the areas that were cropped for representative Western blot images used in Figure 1. Each lane represents one individual animal.

**Supplemental Video S1. Example of EEG traces and simultaneous video recording before, during, and after a spontaneous seizure.** Representative video of electrographic spontaneous seizure with corresponding behavior. The red bars indicate the onset and end of the spontaneous seizure. The spontaneous seizure is characterized by high frequency and high amplitude waves across all EEG electrodes and EMG. The video recording illustrates corresponding behavior during a spontaneous seizure, including rearing, paw waving, bouncing, and loss of posture.
